# Multiple Symmetric Lipomatosis: A Review of 3 Cases

**DOI:** 10.1155/2012/910526

**Published:** 2012-07-16

**Authors:** Emilio Mevio, Michele Sbrocca, Mauro Mullace, Silvia Viglione, Niccolò Mevio

**Affiliations:** Department of Otorhinolaryngology, Ospedale Fornaroli, via Donatore del Sangue, 20013 Magenta, Italy

## Abstract

Multiple symmetrical lipomatosis, or Madelung's disease, is a rare disease of unknown etiology. It is characterized by the presence of loose adipose tissue deposits localized in the cervical region and in the upper body. The neoformations grow slowly and their initial consequence is purely esthetic. They can, however, lead to compression of the laryngotacheal area and of the esophagus. This disease usually affects middle-aged males from the Mediterranean area with a history of alcohol abuse. Although most cases have been sporadic, a few authors have indicated that the disorder may be hereditary. It is thought that this pathology originates from an alteration in lipid metabolism. Since the patients were asymptomatic temperance and diet was proposed, surgical removal of the lipomatose mass is the treatment of choice in case of complications due to fat mass compression on upper aerodigestive tract. The authors present three cases of Madelung's disease with different and particular manifestations.

## 1. Introduction

Multiple symmetric lipomatosis (MSL) is a rare disease also known as Madelung's disease, Launois-Bensaude syndrome, and benign symmetric lipomatosis. The disorder was first described by Brodie in 1846 [1]; Madelung in 1888 and Launois and Bensaude in 1898, characterized the disease [2, 3].

MSL is different from simple obesity, which is characterized by the presence of well-distributed total body fat. In patients affected by MSL symmetric, nonencapsulated fat masses are present in the face, neck, occipital region, and supraclavicular fossa. Fat deposits around the cervical region form a “buffalo hump” and a “horse collar,” while, fat deposits around the parotid region may appear as “hamster cheek.” Fat can penetrate deeply in the surrounding tissues, involving vessels, nerves, and muscles and compressing trachea and esophagus.

The etiology is unknown although autosomal dominant or mitochondrial inheritance has been postulated. However, the great majority of cases of MSL occur in men aged between 30 and 70 years with known alcoholism (in 60/90% of cases). The disorder predominantly affects white males (male : female ratio 15 : 1) of Mediterranean and eastern European populations [4].

The diagnosis is usually easily made on the basis of the history, and clinical appearance. CT or MRI imaging further confirm the diagnosis and value the deeper fatty tissue distribution.

## 2. Cases Presentation

**Case 1**

**Figure 1:**
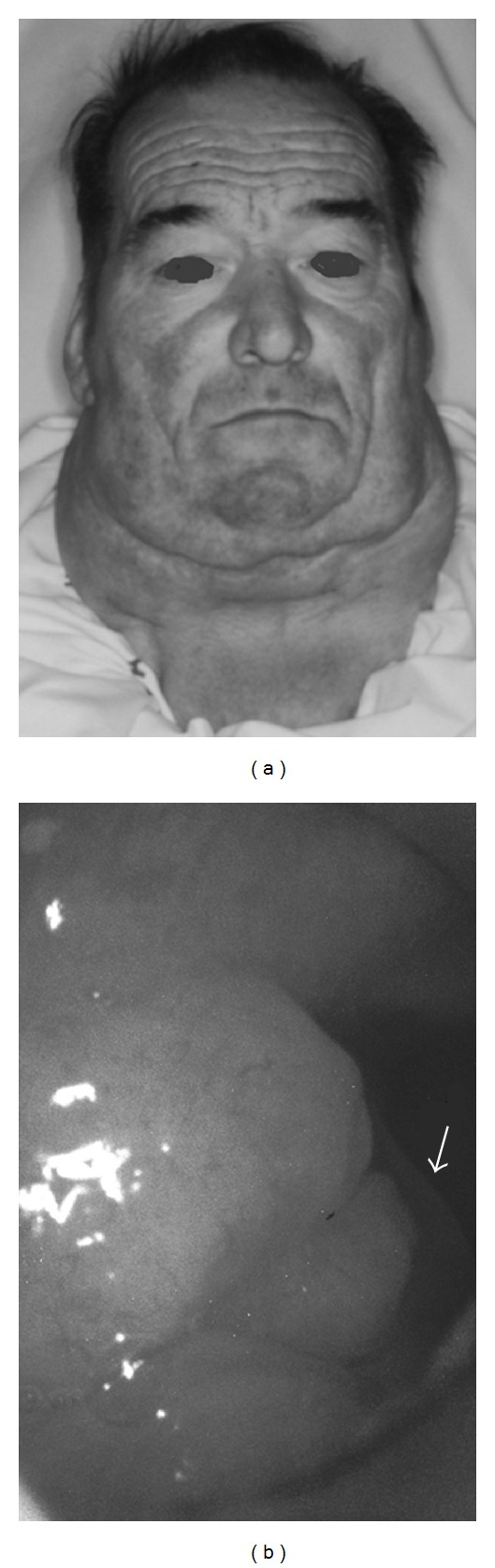
(a) Clinical appearance of subject 1; frontal view with evidence of fatty masses involving parotid region, neck, and upper trunk. (b) laryngoscopic examination revealing fatty infiltration of the left preepiglottic and paralaryngeal space with reduction of the laryngeal vestibular lumen. Left vocal fold (arrow) and glottic space are not involved.

A 65-year-old man presented to our department complaining severe dyspnea and with an 8-year history of a slowly growing fatty mass in the neck, trunk, and upper parts of the arms. The patient began to have difficulty breathing for the last two weeks according to the gradual growth of the mass. The patient had a history of heavy alcohol consumption, altered lipid metabolism, and was a nonsmoker. Laryngoscopic examination revealed fatty infiltration of the left preepiglottic and parapharyngeal space with reduction of the lumen of the laryngeal vestibule determining dyspnea (Figure 1). Computed tomography (CT) scan revealed diffuse, nonencapsulated fatty deposits in the subcutaneous and deeper fascial compartments of the neck, in the mediastinum, and upper trunk. The mass particularly involved the left-superior mediastinum and the laryngotracheal region with compression and dislocation of larynx and superior part of trachea. With a clinical diagnosis of MSL, the patient was taken to surgery. The superficial and deep fatty masses of the neck and trunk were excised. The mass narrowing the laryngeal vestibule was treated with laser surgery through microsuspension direct laryngoscopic approach. After surgery, the respiration of the patient immediately improved.

**Case 2**

**Figure 2:**
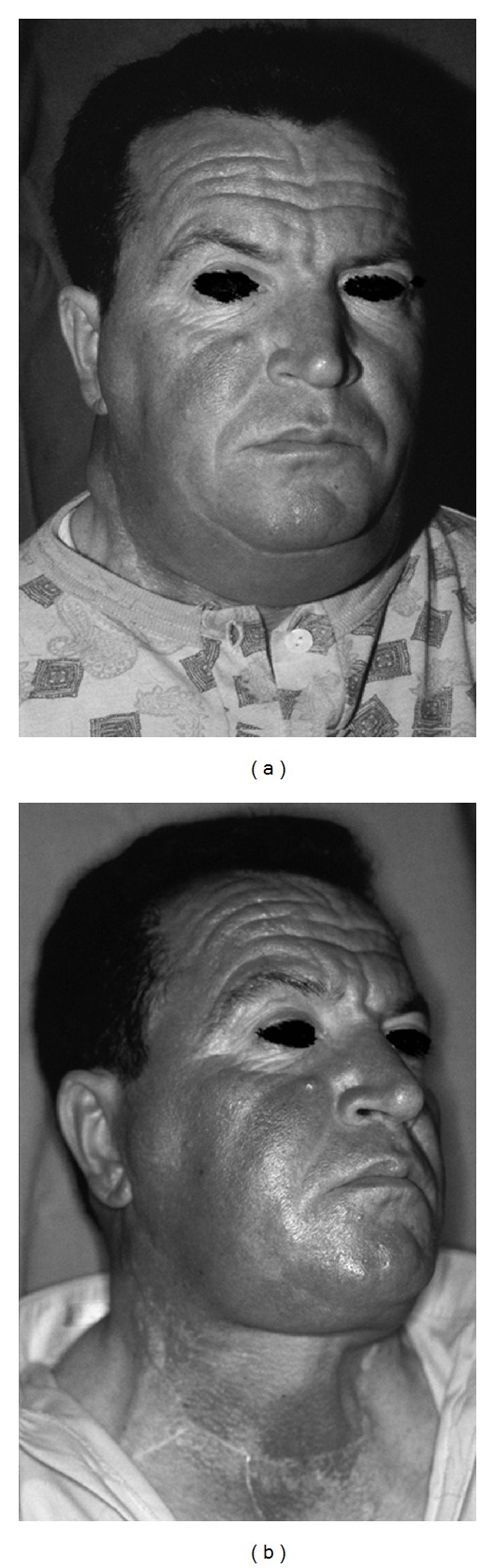
Preoperative view (a), and postoperative view (b) of second patient. The image sequence shows buffalo hump and horse collar disappearance.

A 55-year-old man presented with progressively enlarged masses around his neck for more than 7 years. He had a 25-year history of alcohol abuse. Laboratory examination showed elevated transaminases. Physical examination showed multiple soft masses involving the neck, occipital region, suprasternal, and supraclavear fossa (Figure 2). CT scan showed excess of fat predominantly in the anterior and posterior part of neck and in the supraclavear fossa. The superior mediastinum was partially interested and no sign of trachea or esophagus compression was found. The patient complained about his cosmetic aspect and the reduced range of motion of the head and neck. The patient underwent surgical excision of subcutaneous fat tissue surrounding the neck by one-stage approach (operative specimen weight: 0,920 g).

**Case 3**

**Figure 3:**
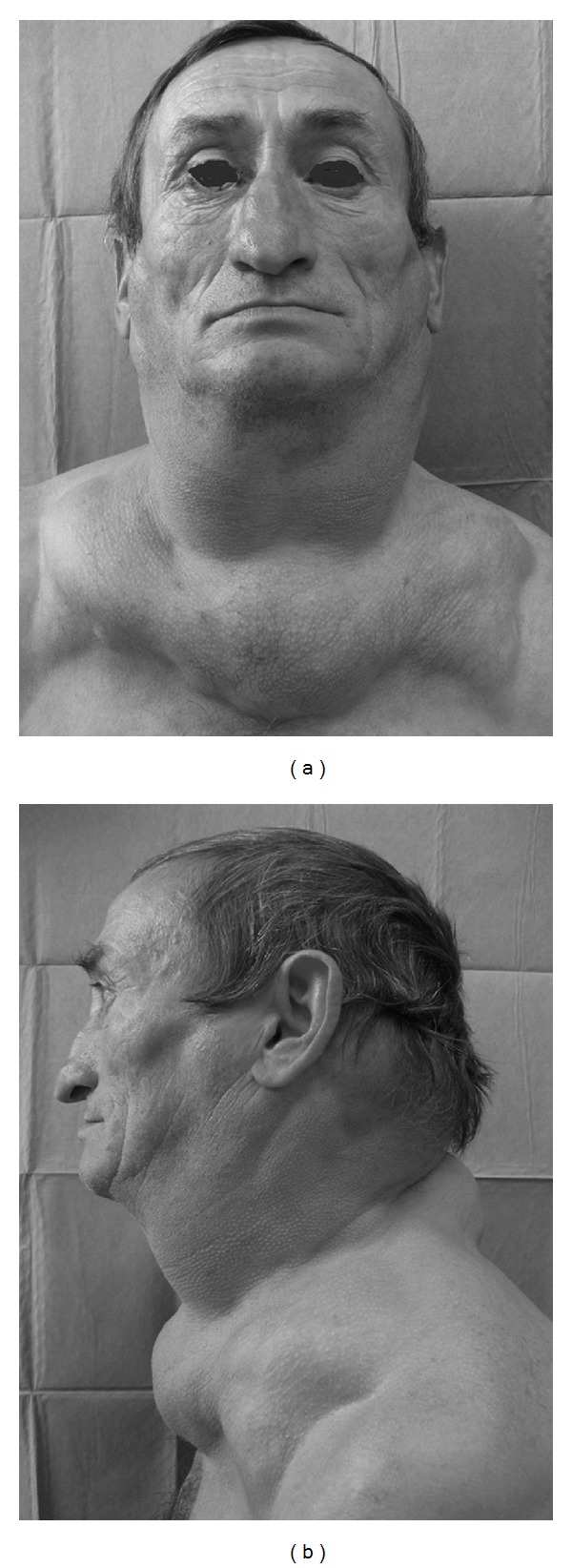
Preoperative frontal and lateral view of subject 3 with evidence of fat masses located in anterior and posterior neck, and upper trunk.

**Figure 4:**
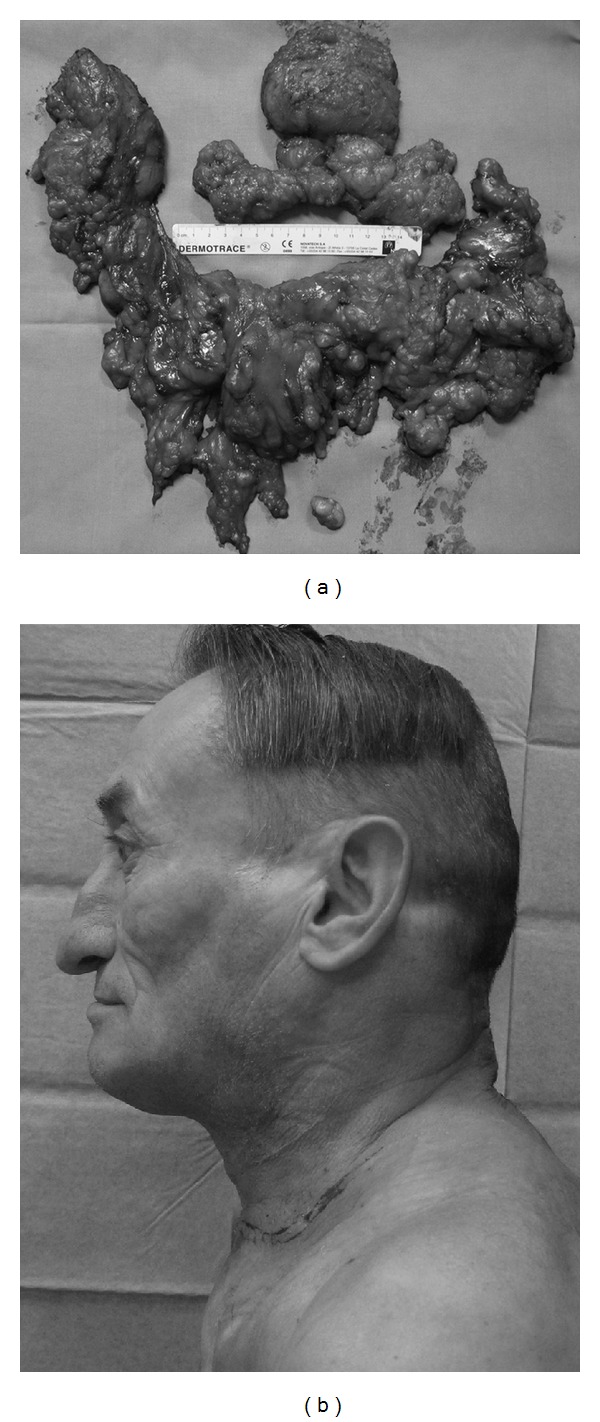
(a) operative specimen of subject 3; about 1180 g of fatty tissue was removed. (b) postoperative profile of the patient showing significant improvement in outward appearance.

A 58-year-old man presented with a 5-year history of a painless, soft, and slow-growing swelling of the neck, upper trunk, upper back, and shoulders (Figure 3). The patient complained of his cosmetic appearance, decreased neck motion, and aerodigestive problems. Patient history was not characterized by alcohol or smoke abuse. CT scan revealed diffuse, nonencapsulated fatty deposits in the mediastinum and in the subcutaneous and deeper fascial compartments of the neck, upper trunk, and back. A clinical diagnosis of Madelung's disease was made. Surgical debulking was performed by one-stage approach: the specimen removed weighed 1180 g (Figure 4). Histological examination revealed mature adipose tissue with an increased fibrocollagenous component.

## 3. Discussion

Two different types of lipomatosis have been identified: familial multiple lipomatosis (FML) and multiple symmetric lipomatosis (MSL). FML is characterized by discrete lipomas that interest the extremities and generally are absent from the neck and shoulders. MSL is a rare disorder marked by the presence of multiple, symmetric nonencapsulated fat masses in the face, neck, upper trunk, and occasionally other areas. Frequently associated findings include hyperlipidemia, hyperuricemia, gout, diabetes mellitus, hypertension, hypothyroidism, liver disease, and polyneuropathies.

The pathogenesis of Madelung's disease is still unknown. Nevertheless, several hypothesis such as defect in the adrenergic stimulated lipolysis, a primary defect within the surface membrane of the adipocyte cell, or a defect in brown mitochondrial DNA (both in inherited and acquired defect) have been proposed [5, 6].

The clinical course of MSL is characterized by a rapid progressing growth of the fat deposits during the early phases of the disease, which thereafter either slowly progresses or remains stable for many years. The long-term lipomatous deposits are often large and cosmetically deforming, furthermore they can cause compression of the upper aerodigestive tract and of great veins. Consequently the patient may complain of dyspnea, dysphagia, and venous stasis in advanced cases [6–10]. Malignant degeneration of fat tissue into myxoid liposarcoma has been reported [11]. 

Alcohol withdrawal and weight reduction are recommended, although they cannot reverse or stop the course of disease. Medical treatment is not effective. Liposuction was proposed as MSL treatment in patients with smaller masses [12]. Surgical debulking is the treatment of choice in patients with a severe cosmetic deformity causing psychological stress and in patients with dyspnea or dysphagia due to compression of aerodigestive tract [13, 14]. 

The cases, described above, characterize three manifestations of the disease. The first one, beside cosmetic appearance, complained of involvement of the left-superior mediastinum and the laryngotracheal region with compression and dislocation of larynx and superior part of trachea. Fat tissue penetrated in larynx reducing the supraglottic space and provoking inspiratory dyspnea. Up till now, only 7 documented cases of direct laryngeal involvement by proliferating adipose tissue in MSL were reported in literature. All the authors proposed the same treatment: a surgical lipectomy in order to decompress the laryngotracheal region and a microsuspension direct laryngoscopic approach to remove the obstructive laryngeal mass [7, 9, 10].

The second patient complained about his cosmetic aspect and was in trouble finding clothes fitting his neck. Because of aesthetic, psychological, and social reasons the patient was submitted to surgical treatment. Abstinence from alcohol was recommended to decrease the rate of recurrence.

The third subject presented the typical picture of the disease with thickened soft tissue in anterior neck, nape, and upper trunk. In this case, there was no history of heavy alcohol consumption, signs of altered lipid metabolism, or other metabolic disorders. The surgery was planned in order to reduce the size of lipomatous masses and patient's inability to move neck and arms freely. 

In the treatment of our patients we did not consider liposuction because of the extension of fatty deposits. Moreover, lipectomy by open surgery is recommended for proper identification of major vessels and nerves and offers the chance of more extensive debulking.
